# Fabrication of Polyamide Thin-Film Composite/Polyethersulfone-Coreshell-Fe_3_O_4_/ZnO Membranes for the Efficient Removal of Pb(II) from Wastewater

**DOI:** 10.3390/membranes15110341

**Published:** 2025-11-17

**Authors:** Nompumelelo Sharol Mbali Kubheka, Muthumuni Managa, Makwena Justice Moloto, Edward Ndumiso Nxumalo

**Affiliations:** 1 Institute for Nanotechnology and Water Sustainability, College of Science, Engineering and Technology, University of South Africa, Roodepoort 1709, South Africa; 57842795@mylife.unisa.ac.za (N.S.M.K.); nxumaen@unisa.ac.za (E.N.N.); 2 Department of Chemistry, Faculty of Applied Sciences, Durban University of Technology, Steve Biko Road, Berea, Durban 4001, South Africa

**Keywords:** nanofiltration membranes, interfacial polymerization, coreshell-Fe_3_O_4_/ZnO nanocomposites, monomers, environmental water

## Abstract

Thin-film composite nanofiltration membranes were fabricated via the interfacial polymerization method from optimized polyethersulfone (PES) mixed matrix membranes, using m-phenylenediamine and trimesoyl chloride monomers, which produced a selective polyamide layer and were used for heavy metal removal. The concentration of trimesoyl chloride (TMC) is a critical factor to govern the properties of the selective polyamide layer, which directly influences the surface morphology and selective performance of (0.5 wt%) PES-coreshell-Fe_3_O_4_/ZnO membranes. Morphological structure, illustrated by SEM images, elucidated the role of TMC addition. FTIR spectra validated the successful formation of the amine and acyl chloride groups. Performance studies illustrated that NF3 (made from 0.1 *w*/*v*% of TMC) showed a unique salt rejection trend (NaCl > Na_2_SO_4_ > MgCl_2_) with an optimal salt rejection of 52.64%, 50.91%, and 12.67%. A low concentration of 0.1 *w*/*v*% of the NF3 membrane was the most optimal high-performance membrane. The adsorption rate of NF3 for Pb(II) ions in real environmental wastewater is attributed to the tailored surface chemistry of the polyamide layered thin-film/PES-coreshell-Fe_3_O_4_/ZnO nanocomposites of the membranes. The maximum Langmuir adsorption capacity at the optimal pH = 5 was 8.8573 mg/g at 25 °C. The fabricated adsorptive nanofiltration membranes alleviated the presence of Pb(II) ions and other competing ions present in environmental wastewater.

## 1. Introduction

Access to high-quality water is increasingly becoming a serious challenge, because wastewater contains undesirable heavy metals. Lead is one of the naturally occurring heavy metals often found in wastewater. Its ductility, malleability, and erosion resistance are some of the physicochemical properties that have made it a popular metal [1,2]. However, its presence in water systems negatively affects human health. For example, it has detrimental effects on human health, such as a disrupted nervous system or gastrointestinal tract, or a malfunction of the kidneys, cardiovascular, hematological, and immunological systems [3,4,5]. Therefore, the removal of Pb from environmental water before being discharged into natural water has become one of the important requirements. A variety of methods, aside from membrane separation technology, have long been employed to remove or reduce heavy metal presence in wastewater, such as coagulation–flocculation, chemical precipitation, oxidation-reduction, and ion exchange. These methods also have frequent drawbacks, such as insufficient removal, costly operating expenses, and the production of hazardous sludges [6,7]. Conversely, adsorption techniques play a critical role as a more economical, user-friendly, and effective option for removing heavy metals from wastewater. Therefore, the fabrication of adsorptive membrane separation techniques would be a more appealing approach. Several researchers [8,9,10] conclusively established various adsorbents that have successfully reduced access Pb(II) ions present in wastewater. The integration of adsorbents into nanofiltration membranes is a visible approach, and the process involves the mechanism of electrostatic repulsion and size–sieving interaction [11]. Unfortunately, the removal of metal ions is not fully achieved due to the insufficient electrostatic repulsion [12].

Limitations of NF membranes, such as low permeability and poor selection of divalent cations, are factors considered by researchers for ideal NF membrane fabrication. Hence, performance optimization of NF membranes has been carried out mainly by the selection of monomers [11,13,14], incorporation of nanoparticles [15,16,17], and adjustment of charge densities [18]. Therefore, modifications of NF membranes are crucial to enhance the performance and morphological strength of NF membranes. To further enhance NF membranes for adsorption processes, researchers have explored composite embedded PES membranes via the inclusion of nanoparticles, interlayers, and surfactants [17,19]. Nanoparticles such as Fe_3_O_4_ and ZnO have been utilized in water applications due to their structural and functional properties that synergistically enhance the selectivity, hydrophilicity, and rejection of PES membranes [20,21]. The incorporation of ZnO nanoparticles has been demonstrated to increase the membrane’s resistance to fouling, which is a common issue in filtration processes [22]. The presence of ZnO helps maintain higher permeate fluxes [23]. The addition of Fe_3_O_4_ may also contribute to the magnetic properties, potentially aiding in the recovery and reuse of the membranes after contamination.

The most common method for producing thin film composite (TFC) nanofiltration membranes is via the interfacial polymerization (IP) method. Diverse types of commercial NF membranes have been produced using the IP method, such as NF270, NF90, NF270 [24,25]. The IP method is based on the reaction of two monomers in a boundary of immiscible solutions. Monomers such as m-phenylenediamine (MPDA), piperazine (PIP), and trimesoyl chloride (TMC) are used to produce TFC nanofiltration membranes [17,26,27]. The addition of these monomers influences the morphological architecture, chemical structure, and performance of the membranes [28]. Regulating the ratio of MPDA/TMC is a necessity, because it results in a looser NF membrane. The NF membranes fabricated using MPDA/TMC have been reported to have satisfactory metal cation rejection attributed to strong steric hindrance [29]. TMC molecules can undergo carboxyl group formation during or after the IP process, causing the membrane to become electronegative in neutral solution settings. Decreasing membrane electronegativity may increase the NF membrane’s heavy metal removal capacity [29]. Therefore, the development of adsorptive PES-coreshell-Fe_3_O_4_/ZnO NF membranes represents a significant technological advancement.

Adsorption of lead removal utilizing different NF membranes has been explored by researchers. Adsorptive TFC Polyamide/PS35 membrane containing cellulose nanocrystals (CNCs), acetylated CNCs (ACNCs), and L-cysteine-modified cellulose nanocrystals (CysCNCs) in the PA layer was examined for the rejection of Pb^2+^ (88.8%, 91.6%, 93.0%, and 95%) [30]. Polyethersulfone membranes were evaluated for the removal of Pb^2+^ [31]. Polyethyleneimine functionalized with multi-walled carbon nanotubes rejected 95% of Pb(II) ions [32]. Polyethersulfone/Fe_3_O_4_@SiO_2_@PrEDAS nano-adsorbent attained 98.5% Pb(II) rejection [33]. A hybrid biosorbent-nanofiltration membrane was utilized to effectively remove Pb(II) from wastewater. It was reported that the biosorbent nanofiltration process successfully removed 98% of Pb(II) ions from wastewater [34]. The above-mentioned research studies illustrate the remarkable potential of adsorptive NF membranes. Therefore, this research study focuses on the fabrication and characterization of adsorptive polyamide thin-film composite/PES-coreshell-Fe_3_O_4_/ZnO nanofiltration membranes, systematically varying the concentration of TMC monomer while keeping the concentration of MPDA constant.

**Scheme 1:**
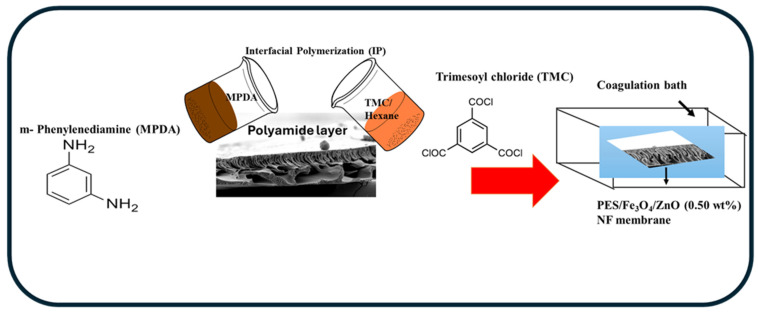
The polyamide/PES-coreshell-Fe_3_O_4_/ZnO nanofiltration membranes fabrication method.

The fabrication of PES MMMs was achieved via the phase inversion process, as detailed in our previous work [35]. The coreshell-Fe_3_O_4_/ZnO nanocomposites embedded onto PES were regarded as a viable method to combat the fouling propensity and trade-off perm-selectivity relationship of membranes, therefore advancing the PES MMMs into polyamide (PA) thin-film composite/PES-coreshell-Fe_3_O_4_/ZnO membranes through interfacial polymerization to enhance their performance and fouling propensity. Monomers such as MPDA and TMC were utilized to fabricate PES-coreshell-Fe_3_O_4_/ZnO nanofiltration membranes because these monomers tend to react rapidly at the interface between aqueous and organic phases, forming a thin layer on the surface of the membranes (Scheme 1). The positions of the functional groups on the aromatic groups enhance the performance of the NF membranes, making these monomer constituents suitable for creating high-performance separation NF membranes. The performance and morphological structure of PA thin-film/PES-coreshell-Fe_3_O_4_/ZnO membranes were determined with the increment of TMC content. This research study focuses on the effect of TMC concentration in tailoring the morphological properties and separation performance of polyamide thin-film composite/PES-coreshell-Fe_3_O_4_/ZnO membranes. This study, therefore, aims to fabricate polyamide thin-film composite adsorptive PES membranes for the efficient removal of Pb(II) from environmental wastewater. The novelty of this study is that it utilizes TMC concentration to engineer the polyamide selective layer that synergistically combines rejection and adsorption functionalities, enabling efficient Pb(II) removal from complex real wastewater.

## 2. Materials and Methods

Polyethersulfone (MW = 58,000 g/mol), *N*-methyl-pyrrolidone (NMP), polyvinylpyrrolidone (PVP) (MW = 40,000 g/mol), *m*-phenylenediamine (MPDA, 99.0%), trimesoyl chloride (TMC, 98%), sodium anhydrous n-hexane (99.0%), sodium dodecyl sulfate (SDS, 99.0%), sodium sulfate (Na_2_SO_4_), sodium chloride (NaCl), magnesium chloride (MgCl_2_), hydrochloric acid (HCl), and sodium hydroxide (NaOH) were used to adjust the pH of the solutions. All chemicals were purchased from Merck (Johannesburg, South Africa) and used without any further treatment.

### 2.1. Preparation of PES/Fe_3_O_4_/ZnO Nanofiltration Membranes

The PES membranes were fabricated using the interfacial polymerization method adopted from [36]. The synthesis of the embedded nanocomposites and the optimum PES/Fe_3_O_4_/ZnO membranes were prepared as per previously reported studies [35,37]. The prepared MMMs of PES/Fe_3_O_4_/ZnO were pretreated by immersing the membranes into deionized water containing 0.50 wt.% of sodium dodecyl sulfate (SDS) overnight. SDS plays a distinct role in the fabrication of NF membranes because it alleviates the aggregation of nanocomposites onto the surface of the membranes [38]. The membranes were further immersed in deionized water for 1 h and then immobilized with a tape onto a glass plate and allowed to dry in the oven at 60 °C for 2 h. Thereafter, 150 mL of deionized MPDA solution was prepared, and then the dried membranes were soaked in MPDA solution for 2 min. The prepared membranes were further immersed into TMC in n-hexane solution for 30 s. Different concentrations (0.1, 0.2, 0.3 *w*/*v*%) of TMC dissolved in 150 mL of n-hexane solution were varied. The functionalized membranes were dried at 60 °C for 3 min and then stored in deionized water. Table 1 depicts the different compositions of the prepared membranes referred to as NF0–NF5.

### 2.2. Collection and Analyses of Wastewater Samples

Wastewater samples were collected from a wastewater treatment plant located in the Vaal region (Gauteng, South Africa). The water samples were collected from the biological nutrient removal (BNR) process using 250 mL plastic bottles. The samples were acidified and placed in cooler bags, transported to the laboratory, and stored in a cold room set at a temperature of 4 °C. The samples were allowed to equilibrate at room temperature before the commencement of analysis. The physicochemical properties of the collected wastewater samples are detailed in Table 2.

### 2.3. Characterization Techniques

#### 2.3.1. Physiochemical Analysis

Scanning electron microscopy (SEM, JEOL JSM-IT300 (JEOL Ltd., Tokyo, Japan)) was used to study the surface and cross-sectional morphological properties of the membranes. The hydrophilicity of the produced membranes before fouling and after fouling was investigated using contact angle measurements (sessile drop method). Topographic images of the membranes were measured by AFM. The functional groups of PES and PES/PVP membranes were confirmed using the ATR PerkinElmer FTIR spectrometer Frontier (PerkinElmer, Waltham, MA, USA) (spectrum 100 spectrometer). Filtration studies were conducted using dead-end cells with an effective membrane area of 0.00126 cm^2^, and the membranes were cut circularly to the cell surface area. The pure water flux measurements were carried out at 5–10 bars. The water flux was calculated using Equation (1), and the mean pore radius was calculated using Equation (2).

(1) $$Jw=\frac{V}{A∆t}$$

where *Jw* is the water flux (L·m^−2^·h^−1^), the volume of water permeation flux is *V* (L), the effective membrane area is *A* (m^2^), and the sampling duration (h) is ∆*t*.

(2) $$\varepsilon =\frac{W_{w}-W_{d}}{A\times Ɩ\times d_{w}}\times 100$$

where the gravimetric mass of the membranes is *W_w_* and *W_d_*, the effective membrane area is *A* (m^2^), the thickness of the membranes is l (m) and *d_w_* is the density of water. The average pore size was calculated using Equation (3).

(3) $$r_{m}=\sqrt{\frac{\left(2.9-1.75\varepsilon \right)8ղlQ}{\varepsilon A∆P}}$$

where ղ (8.9 × 10^−4^ Pa·s) is the water viscidity, the thickness of the membranes is *l* (m), the volume flow speed (m^3^/s) is *Q*, the effective membrane area is *A* (m^2^), and ∆*P* is the operational compression (MPa).

#### 2.3.2. Rejection Studies

Salt rejection of nanofiltration membranes was assessed using the dead-end filtration setup at the salt concentrations (NaCl, MgCl_2_, Na_2_SO_4_) of 2000 ppm at pH (6.88, 6.04, and 7.08) for the salts under the operating pressure of 6 bar. The electrical conductivity of the salt concentration was measured using the Consort C6010 conductivity meter (Consort, Turnhout, Belgium).

Lead rejection experiments from environmental wastewater samples were conducted using the same dead-end filtration system used in water permeability tests. UV–visible Spectroquant Pharo300 (Sigma-Aldrich, St. Louis, MO, USA) was used to determine the concentrations of the heavy metals. Equation (4) was used to calculate metal ion retention (R):

(4) $$R\;(\%)\;=\;(1\;-\;\frac{C_{p}}{C_{f}})\times 100$$

where the concentration of the permeate is C_p_ and C_f_ is the concentration of the feed solutions (ppm). The concentration of permeate ions was determined before and after the filtration process.

#### 2.3.3. Performance Studies

Antifouling studies of the membranes were determined using the permeate of foulants (J_f_) (environmental wastewater samples). The pH of environmental water samples was measured and obtained as pH = 7. All membranes were backwashed with deionized water at 6 bars after the filtration of the feed water. The membranes were immersed in EDTA (10 mM) as a chelating agent for 60 min, thereafter backwashed using deionized water, and were measured at 6 bars. These membranes were examined for three cycles. The flux recovery was determined using Equation (5).

(5) $$\mathrm{FRR}\;(\%)=(\frac{{Jw}_{2}}{{Jw}_{1}})\times 100$$

The mass transport resistance of the foulants (environmental wastewater samples) was determined using the reversible (R_r_) fouling ratio, irreversible (R_ir_) fouling ratio, and total fouling ratio (Rt) Equations (6)–(8).

(6) $$R_{r}\;(\%)=(\frac{{Jw}_{2}-JF}{{Jw}_{1}})\times 100$$

(7) $$R_{\mathrm{ir}}\;(\%)=(\frac{{Jw}_{1-}{Jw}_{2}}{{Jw}_{1}})\times 100$$

(8) $$R_{t}\;(\%)=(1-\frac{JF}{{Jw}_{1}})\times 100$$

#### 2.3.4. Adsorption Studies

Batch adsorption studies using spiked environmental water under ambient conditions and temperature were performed. The concentration of Pb(II) from the collected real wastewater samples was below the detection limit of the analytical instrument. A stock solution of 30 mg/L lead nitrate was prepared and used to spike environmental water samples. The effect of pH for the spiked environmental water samples was studied by varying pH 3–7, which interacted with 20 mg of PA TFC/PES-Fe_3_O_4_/ZnO (0.05 wt.%) membrane. The effect of concentration was evaluated by varying 5 mg/L to 30 mg/L of the stock solution into 25 mL of environmental water. The abovementioned parameters were allowed to shake for 24 h. Thereafter, the effect of time was further studied for 30 min to 210 min. After adsorption, the environmental samples were analyzed for the characterization of metals utilizing NexION 350 ICP-MS, PerkinElmer SA (Pty) Ltd. (Midrand, South Africa). The removal percentage efficiency of lead (II) ion was calculated by using Equation (4). Both Langmuir (Equation (9)) and Freundlich (Equation (10)) isotherm models were fitted into nonlinear models.

(9) $$qe=(\frac{QmaxKlCₑ}{1+KlCₑ})$$

(10) $$qₑ=k_{f}\cdot {Cₑ}^{\frac{1}{n}}$$

#### 2.3.5. Desorption Studies

Desorption of NF membranes was employed using EDTA (10 mM). The membranes were immersed in 25 mL of EDTA solution and allowed to shake for 60 min, and were further washed with DI water. Thereafter, the membranes were immersed into 25 mL of optimized Pb(II) solution containing environmental wastewater samples. The solutions were allowed to shake for 90 min and, thereafter, analyzed using ICP-MS. The desorption–adsorption cycles were repeated three times. The desorption efficiency was determined using Equation (11).

(11) $$\mathrm{Desorption}\;\mathrm{efficiency}\%=(\frac{Released\;Metal\;ion\;concentration}{Initial\;Adsorbed\;metal\;ion\;concentration\;})\times 100$$

## 3. Results and Discussion

### 3.1. Morphological and Topographical Studies of PES-Fe_3_O_4_/ZnO Nanofiltration Membranes

**Figure 1:**
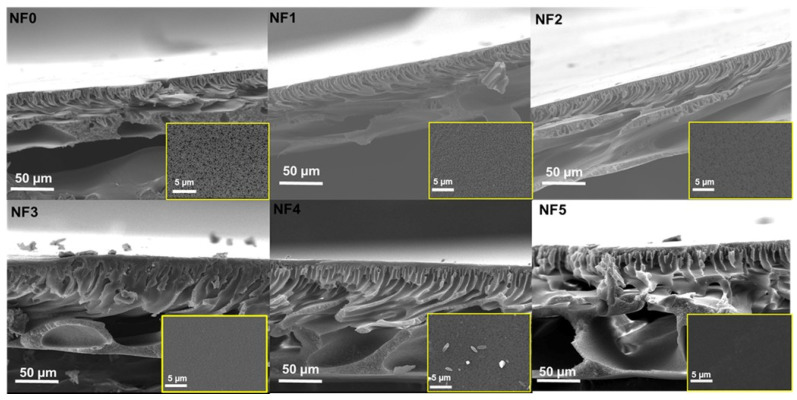
Surface and cross-section SEM images of PA TFC/PES-coreshell-Fe_3_O_4_/ZnO nanofiltration membranes.

**Figure 2:**
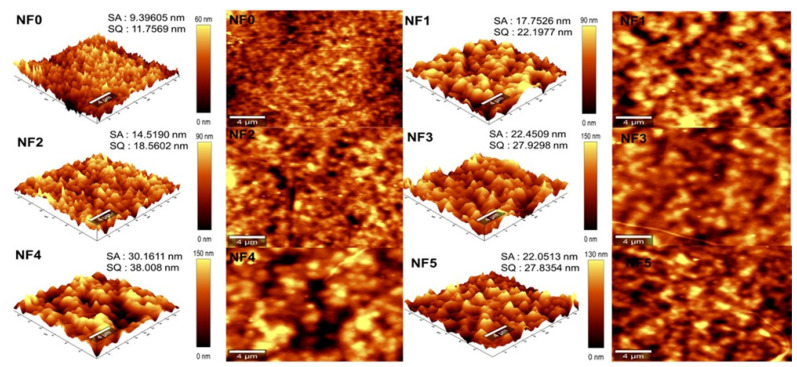
Two- and three-dimensional topographic AFM images of PA TFC/PES-coreshell-Fe_3_O_4_/ZnO nanofiltration membranes.

The surface and cross-section morphological structure (Figure 1) and 2D-topographical images (Figure 2) of the membranes illustrate that all the membranes were porous, which is directly crucial to facilitate the high permeance, selectivity, and mechanical integrity of the membrane. Contrary to the ridge-and-valley-like morphology attributed to amine monomers reported by researchers [39,40], the surface morphology of all the PA membranes exhibited semi-smooth porous morphology. This unique observation demonstrates that the monomer diffusion and polymerization reaction are altered by the presence of Fe_3_O_4_/ZnO nanocomposites that are underneath the selective PAlayer. The Fe_3_O_4_/ZnO nanocomposites of NF3 are visible on the surface of the membranes protruding through the selective PA layer, exhibiting rod-like nanocomposites providing availability of adsorptive sites that enhance membrane functionality. Therefore, understanding the unique observed surface layer is insufficient from just the surface results.

The cross-section morphologies of all the membranes showed a thin, dense polyamide layer on the top surface, demonstrating the successful formation of the selective layer governing the solute separation. While maintaining the finger-like morphologies with interconnected tunnel channels, macrovoids that facilitate high permeate flow, the mechanical integrity was preserved. The consistent observation of the asymmetric features from the cross-section was attributed to the phase inversion process detailed previously [35]. Distinct Fe_3_O_4_/ZnO nanocomposites on the top surface and tunnels of NF3 were observed, as was a spongey layer at the bottom. Furthermore, NF0, NF4, and NF5 were observed to have a spongey layer at the bottom surface of the membranes, except for NF2.

Generally, the increment in surface roughness could result in more adhesion between the modified membrane surface and the contaminates, enhancing permeability or resulting in sufficient membrane fouling. The 2D and 3D AFM images of PES-Fe_3_O_4_/ZnO nanofiltration membranes were analyzed using the AFM technique. The surface roughness (Sa) of NF0 was as low as 9.3960 nm compared to the other NF membranes. It is noticed that the surface roughness of the membranes increased with the addition of PVP, with a solvent evaporation time of 180 s, as reported in the previous study [35], as well as an increment of TMC concentration. The intensified brightness and dark ridge-valleys of the membranes illustrate that NF membranes’ surface roughness can be influenced by the different optimized parameters (PVP concentration, 180 s EVT) and the different concentrations (0.1, 0.2, 0.3 *w*/*v*%) of TMC monomer. The observed findings corresponded with other studies [41,42]. NF4 has the highest Sa of 30.1611 nm compared to NF3, which had an Sa of 22.45 nm. This illustrates that the surface roughness of NF3 was smoother, with enhanced hydrophilic properties, suggesting that it might be less prone to fouling [43]. It was further noticed that NF5 had a lower surface roughness of 22.0513 nm compared to NF4, indicating that NF5 might be the maximum hydrophilicity of the membranes due to the incremental loading of TMC monomer on the surface layer of the membranes. Ultimately, this is expected to affect the performance of the NF5 membrane due to mechanical integrity, corresponding to the reported literature studies [28,43]. Therefore, TMC loading affected the morphological and topographical architecture of the membranes.

### 3.2. Hydrophilic Properties of PES-Fe_3_O_4_/ZnO Nanofiltration Membranes

**Figure 3:**
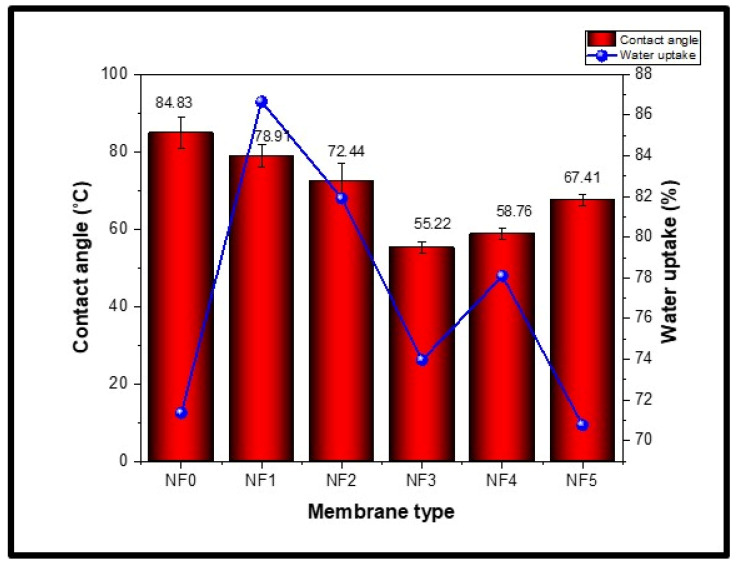
Contact angle and water uptake of PA TFC/PES-coreshell-Fe_3_O_4_/ZnO nanofiltration membranes.

Contact angles are used to assess membrane hydrophilicity, with lower contact angles indicating higher hydrophilicity, enhanced water permeability, and fouling propensity [44,45]. Figure 3 shows the contact angles of NF0–NF2 that are observed to be close to 90°, which implies that the surfaces of these membranes hinder wettability, suggesting that the membranes are less hydrophilic. As the content of TMC loading increased from NF3 to NF5, it was observed that the membranes became more hydrophilic. However, NF3 was observed to be highly hydrophilic at 55.22°. This further indicates that the membranes decrease hydrophilicity at the highest loading of TMC content. It was observed that the higher water uptake implied that the membranes were hydrophilic, supporting the contact angle findings. The water uptake increased after the addition of the pore former PVP, and significantly decreased as the solvent evaporation time was 180 s. It was observed that, as the TMC (0.1, 0.2, 0.3 *w*/*v*%) concentrations increased, there was a significant decrease in the water uptake, further impacting the hydrophilicity of the membranes. Therefore, there was a correlation between the contact angles and water uptake of the membranes. The membranes with low TMC content have greater hydrophilicity, which demonstrates that NF3 can eliminate high multivalent ions; these findings are consistent with [41].

### 3.3. Porosity and Pore Size

**Figure 4:**
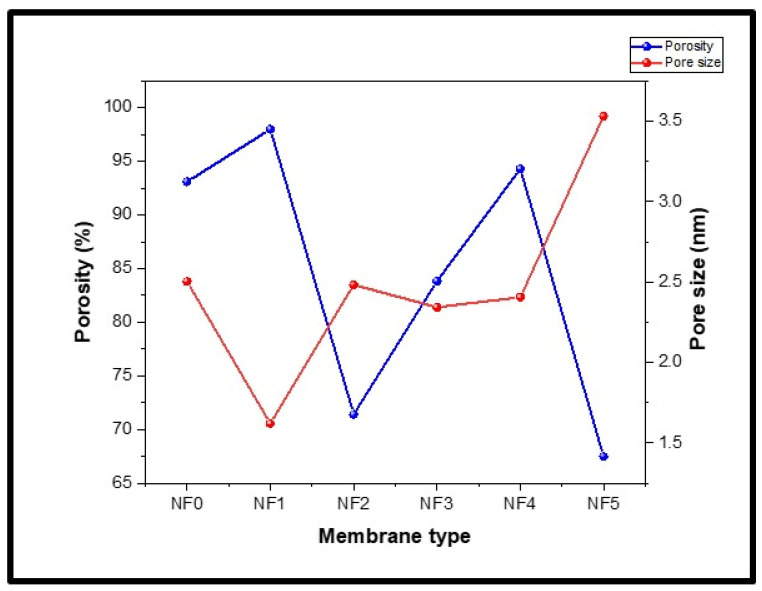
The porosity and mean pore radius of PA TFC/PES-coreshell-Fe_3_O_4_/ZnO nanofiltration membranes.

**Figure 5:**
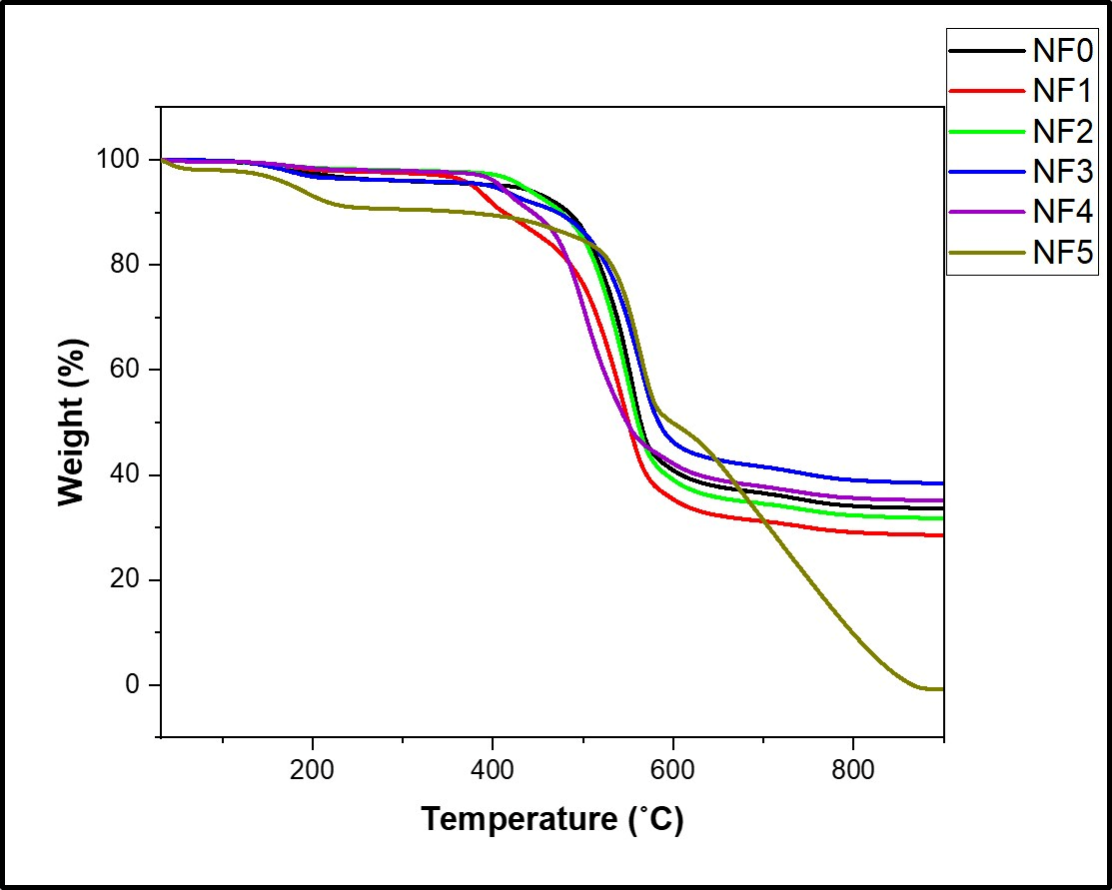
Thermogravimetric graphs of PA TFC/PES-coreshell-Fe_3_O_4_/ZnO nanofiltration membranes.

The percentage of the membrane volume filled by pores is referred to as porosity. It is known for its mechanical stability and water flux. The size of the pores dictates the size of the solutes that can pass through, and it affects the rejection performance of the membranes. These two parameters indirectly interlink with the membrane structure. Figure 4 shows that NF0, NF1, and NF4 had high porosity and pore sizes of 97.87% (2.49 nm), 92.93% (1.61 nm), and 94.32% (2.40 nm). It is further observed that NF3 and NF5 had a porosity of 83.84% (2.33 nm) and 67.50% (3.53 nm). Meanwhile, the addition of TMC concentration generally shows a decrease in porosity, and the relationship with pore size is complex. The deviation of NF5, having a high mean pore size of 3.53 nm despite its porosity, contradicts the expected formation of a denser polyamide layer with smaller pores. This apparent contradiction is due to the polyamide structure formed at higher TMC concentration loadings. The morphological (Figure 1) and thermal (Figure 5) analyses indicate that NF5 has structural defects or irregularities and an overly stretched decomposition stage. The defects result in a polyamide layer structure, characterized by a highly cross-linked polymer matrix that contributes to the low porosity, alongside micro-voids that are measured as a large mean-pore size. The results obtained directly correlate to the morphological and topographic structure of the membranes, further corresponding with findings of the contact angle (Figure 3). The determined pore sizes are within the polyamide matrix, demonstrating that the effect of increasing the monomer concentration (TMC) during interfacial polymerization led to the reduction in water permeance and altered the pore size, while longer reaction times decrease permeance without significantly changing the pore size of the membranes [46]. Since NF3 has visible nanocomposites on the surface layer, less surface roughness, smaller pore size, and a higher contact angle, it is expected that this membrane might have high selectivity for contaminant removal.

### 3.4. Thermal Properties of NF Membranes

Thermal degradation of NF membranes was performed using a TGA 5500 Discovery series (TA instruments), as shown in Figure 5. The measurements were conducted at an ambient temperature of 900 °C. The initial decomposition stages of all the NF membranes were ~3%, which was attributed to water decomposition. The decomposition stages observed for NF0 were due to moisture at 96.29 °C, and the first decomposition stage at 219.43 °C to 484.04 °C was due to thermal degradation of the sulphone group (-SO_2_) of PES that caused the backbone of the PES polymer chain to break down. The second decomposition stage was observed at 574.29 °C to 732.98 °C due to aromatic ether linkages (-O-) groups in the backbone of the PES. The NF2 membranes were observed to have the first decomposition stages at 219.43 to 406.48 °C due to PVP decomposition. This shift in PES decomposition to 593.73 °C suggests that the addition of PVP enhances the thermal stability of the membranes, due to the hydrogen bonding that alters the thermal stability of the membranes. NF0, NF1, and NF2 membranes were observed to have a thermally stable final decomposition stage. Membranes of NF3, NF4, and NF5 contained incremental loadings of TMC, and it was observed that the thermal stability of the membranes for NF3 and NF4 was stable compared to NF5. The first thermal decomposition of NF3, NF4, and NF5 was observed from 211.8 to 498.91 °C, 219.43 to 463.61 °C, and 242.56 to 465.80 °C, due to the PVP addition that altered the stability of the membranes. The second decomposition stage was observed at 498.91 to 609.60 °C, 463.61 to 556.04 °C, and 465.80 to 585.33 °C, and it was due to the PES molecular backbone (sulphite and phenolic groups). The third decomposition stage was observed at 609.60 to 686.11 °C, 556.04 to 680.41 °C, and 585.33 to 638.76 °C, due to the decomposition of Fe_3_O_4_/ZnO nanocomposites, although NF5 had been shown to have a distinct thermal instability, since it had a further fourth decomposition stage that was overly stretched at 638.76 to 832.31 °C, which was attributed to the high loading of TMC. This illustrates that there might be morphological irregularities in the membrane. Therefore, all membranes except for NF5 had a final decomposition stage >−500 °C residue due to carbonization and other inorganic components, and the NF5 decomposition stage was observed at >600 °C.

### 3.5. Surface Chemistry of PES-Fe_3_O_4_/ZnO NF Membranes

**Figure 6:**
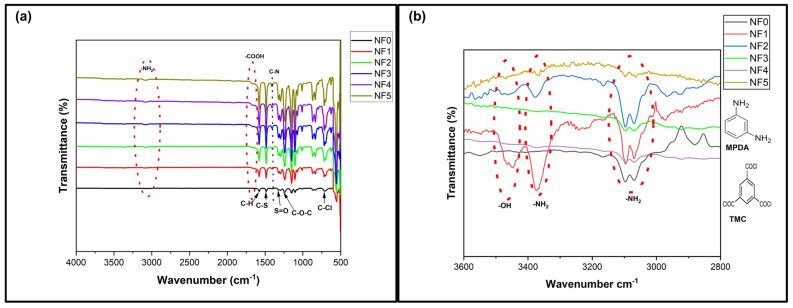
FTIR spectra of PA TFC/PES-coreshell-Fe_3_O_4_/ZnO nanofiltration membranes (**a**) full range spectra and (**b**) expanded FTIR Spectra 3600 to 2800 cm^−1^.

The chemical components of PES-Fe_3_O_4_/ZnO NF membranes were studied using FTIR spectroscopy (Figure 6). The spectrum for all membranes confirmed the presence of PES, indicated by the characteristic peak at 1580 cm^−1,^, attributed to the C-H stretching vibration of the benzene ring. A peak at 1479 cm^−1,^, attributed to the C-S bond stretching, was obtained. The aromatic ether (C-O-C) stretching bond was observed at 1296 cm^−1^. Furthermore, peaks at 1323 cm^−1^ and 1149 cm^−1^ of NF membranes showed the SO_2_ group’s symmetric and asymmetric stretching vibrations, respectively [35]. Additionally, the presence of Fe_3_O_4_/ZnO nanocomposites was confirmed by metal oxide peaks at 454.17 and 592.88 cm^−1^ [35]. The successful formation of the selective polyamide layer through the interfacial polymerization process was confirmed. The peak at 1409 cm^−1^ was observed, which is attributed to amide bonds (C-N, stretching vibrations), confirming the presence of MPDA, and the peak at 717 cm^−1^ was attributed to the C-Cl stretching bond, signifying the presence of TMC. The presence of the broad O-H group peak at 3449 cm^−1^ for NF1–NF2, attributed to PVP additives, and a distinct peak at 3374 cm^−1^, was observed for NF1, NF2, NF4, and NF5, which is attributed to the presence of amine functional groups from MPDA. By contrast, the absence of this amine peak in NF3 suggests a complete reaction of polyamide surface chemistry of the monomers, potentially attributed to N-chlorination and hydrolysis of the NH_2_ groups. The distinct chemical alteration correlates with the enhanced hydrophilicity of NF3 with a contact angle of 55.22° (Figure 3), compared to the other membranes [46,47,48]. The smoother surface of NF3, as observed in AFM (Figure 2), could be attributed to the resulting polyamide surface chemistry. Higher loadings of TMC constitute instability of the membrane, therefore resulting in the decrement of the hydrophilicity of the membranes. The obtained FTIR results confirmed the successful formation of TFC membranes.

### 3.6. Performance Evaluation for PES-Fe_3_O_4_/ZnO Nanofiltration Membranes

#### 3.6.1. Permeation Performance

**Figure 7:**
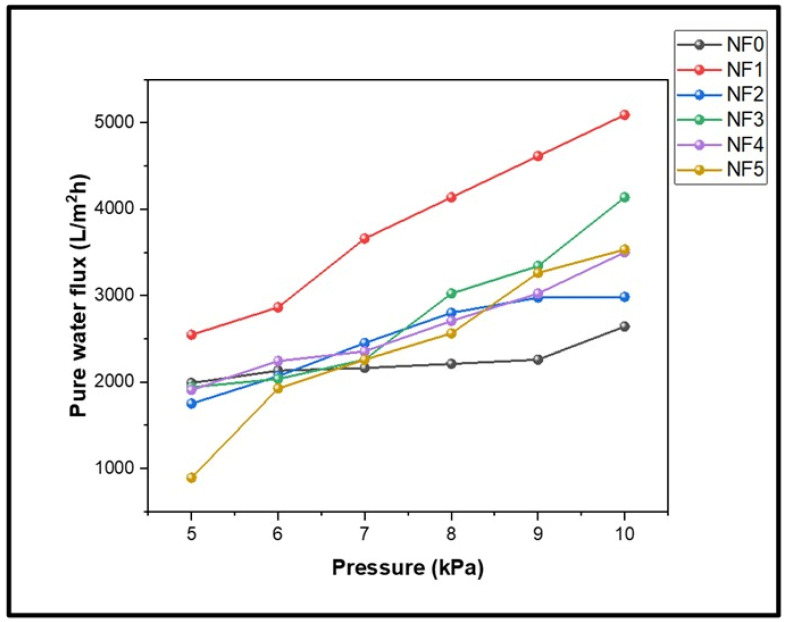
Pure water flux of PA TFC/PES-coreshell-Fe3O4/ZnO nanofiltration membranes.

Owing to the high hydrophilic properties of the modified membrane made by the coating method, these membranes are supposed to have greater water flux than the modified membranes made by the IP method. Modified membranes fabricated using the IP method are generally supposed to have high permeation compared to the pristine membrane. According to this study (Figure 7), it was observed that NF2 and NF5 had higher permeation flux compared to the other membranes. This implies that the hydroxyl groups from the addition of PVP and the increment in TMC significantly enhanced the permeation flux and hydrophilicity of the membranes. The enhancement might be attributed to a large number of hydroxyl groups, amine groups, and carboxylic groups present on the thin layer of the PES-coreshell-Fe_3_O_4_/ZnO nanofiltration membranes compared to the pristine (NF0) [35]. The coreshell-Fe_3_O_4_/ZnO composites contain a large number of hydroxyl (-OH), amino groups (-NH_2_), and carboxylic (-COOH) groups, which can give unique properties to the membrane, altering its morphology and directly improving the flux and membrane permeability. Additionally, monomers facilitate the IP process and also enhance the performance of the membranes. As a result, the presence of all these hydrophilic functional groups facilitates and accelerates the transportation of water through the channels of the membranes, directly influencing the morphological architecture and permeation rate of the membranes [20,48,49,50].

#### 3.6.2. Rejection of Na_2_SO_4_, NaCl_,_ and MgCl_2_ Salts

**Figure 8:**
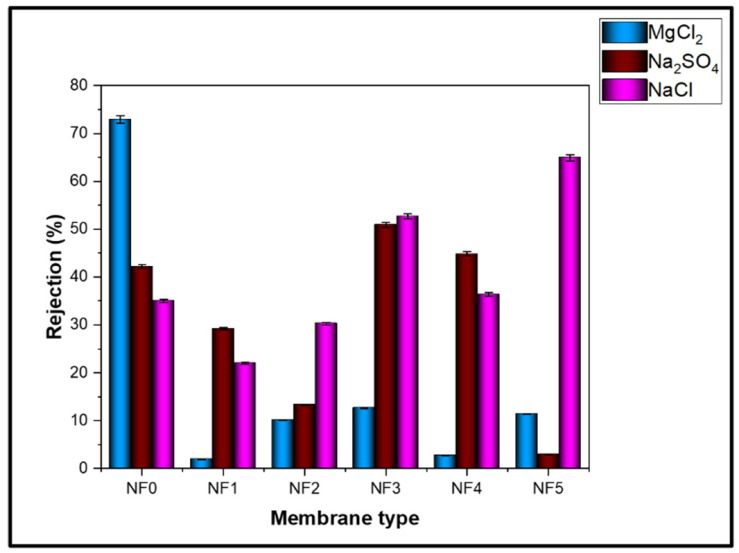
Salt rejection of MgCl_2_, Na_2_SO_4,_ and NaCl for PA TFC/PES-coreshell-Fe_3_O_4_/ZnO nanofiltration membranes.

Studies have reported a common salt rejection trend of NF membranes in the following order: Na_2_SO_4_ > MgCl_2_ > NaCl [51,52]. The observed trend is generally associated with membranes that have a negatively charged membrane surface. Membranes with a negatively charged surface reject divalent anions and monovalent cations favorably [29,53]. The variation in concentration of TMC dosage, especially at lower concentrations, affects the charge property of the membrane, improving the selectivity of the membrane [29]. Figure 8 shows that there was no consistent salt rejection trend for all NF membranes (NF0–NF5) pressured under 6 bars. The NF0 showed high MgCl_2_ (72.91%) rejection, illustrating that the selective polyamide layer enhanced the rejection of MgCl_2_. NF1 and NF2 showed relatively low rejection of salts, demonstrating that the PA layer or membrane morphologies might have defects. Meanwhile, NF5 showed a high rejection of 64.93% for NaCl, contrary from the general reported salt rejection for negatively charged NF membranes. Noticeably, NF3 had high rejection of Na_2_SO_4_ (50.91%) > NaCl (52.64%) > MgCl_2_ (12.67%). This is attributed to the surface chemistry of the incorporated coreshell-Fe_3_O_4_/ZnO nanocomposites within the polyamide layer. This observed anomaly can be attributed to the hydrophilic coreshell-Fe_3_O_4_/ZnO nanoparticles that alter the selective layer [54]. Although zeta potential measurements could provide confirmation on the contaminant removal, the observed salt rejection trends suggest that there was an alteration in the membranes’ surface charge properties. Therefore, the effect on the mechanistic NF membranes is due to the size exclusion, Donnan exclusion [55], and ion interaction [56]. The low rejection of MgCl_2_ indicates that the membrane may have a reduction in the negative charge density that permits the passing of Mg^2+^ cations [52,57]. Meanwhile, the rejection of NaCl and Na_2_SO_4_ is governed by the hydrated radii and steric effects within the altered polyamide layer. The size exclusion mechanism overrides the charge-based rejection due to the presence of the coreshell-Fe_3_O_4_/ZnO nanocomposites. This emphasizes that all influential optimized parameters from our reported work [35], and the nanocomposites layered underneath the selective polyamide layer, contribute to the separation performance of membranes.

#### 3.6.3. Physicochemical Characterization of Environmental Wastewater Samples

Table 2 shows the pH, conductivity, DO, COD, and TDS of the collected wastewater. The pH of the wastewater sample was within the optimal pH range of wastewater, while other physicochemical properties were measured to be lower than the standards of the Department of Water Affairs and Forestry and the Green Drop Certification of South Africa [58,59]. The low oxygen levels indicate that the microbial process necessary for decomposing organic matter may be impeded. This high COD content observed further indicates that there is poor aeration, possibly due to dysfunctional wastewater treatment filters, therefore compromising the regulatory standard required from the wastewater treatment plant.

#### 3.6.4. Heavy Metal Ions Rejection and Fouling Propensity of the Prepared Membranes

**Figure 9:**
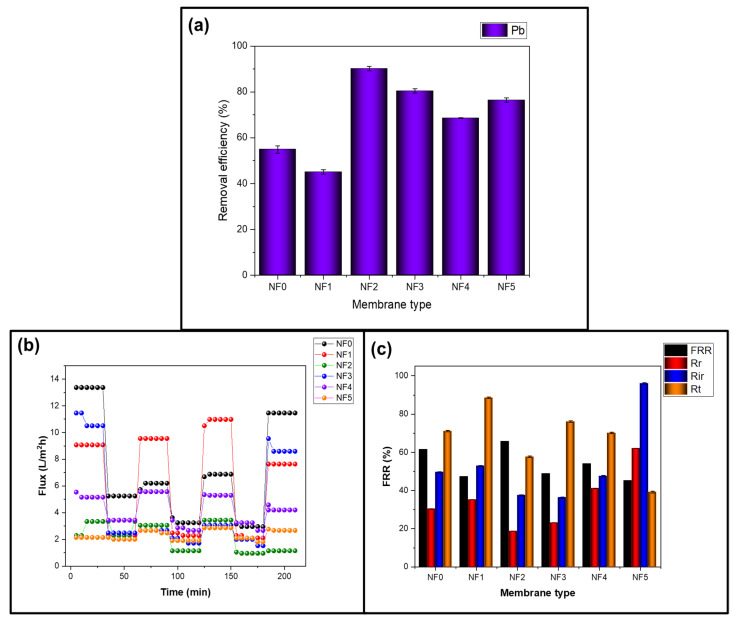
(**a**) Rejection of Pb(II) using the prepared membranes, (**b**) fouling propensities, and (**c**) flux recovery ratios of the membranes.

The Spectroquant photometric method was used to analyze the removal of Pb(II) ions from environmental wastewater. It is important to note that the Spectroquant method is a colorimetric technique that is susceptible to complex interference from complex wastewater constituents, such as COD and other ions. The results of Pb(II) removal for NF membranes in Figure 9a illustrate that NF2 and NF3 had the highest Pb(II) removal, despite the absence of the nanocomposites in NF2; the optimized surface properties from the phase inversion process and the selective polyamide layer provide ample active sites to facilitate adsorption and sieving. It is observed that unmodified NF membranes have relatively low Pb(II) rejection compared to the modified membranes. This is attributed to the adsorptive properties associated with coreshell-Fe_3_O_4_/ZnO nanocomposites due to the presence of excess polar amine groups that interact more with Pb(II), enhancing the adsorptive sites on the membrane surface. Among the modified membranes, NF3 showed the highest removal of Pb(II) (80.39%), containing 0.1 *w*/*v*% TMC loading. Table 3 shows different NF membranes that were enhanced with different concentration loadings of TMC and their effects on heavy metals and salt rejection. Therefore, a reasonable trade-off relationship between permeability and Pb(II) rejection was illustrated by the produced modified NF membranes. Figure 9b shows the filtration cycles of NF membranes after 210 min. All membranes showed a flux decline after every recovery cycle; the recovery cycles are subsequently stable for all the membranes. Noticeably, the flux filtration cycles of NF0 and NF1 are consistently high compared to NF2 membranes, and modified NF membranes (NF3–NF5) showed a low filtration rejection cycle of environmental samples. This is attributed to the clogged pores on the membrane surface due to the presence of nanocomposites after prolonged filtration cycles [60]. Figure 9c shows the antifouling properties of the prepared membranes. The FRR of 61.50% for NF0 and 65.74% for NF1 was higher than other membranes, indicating these membranes favored reversible fouling [61]. All membranes were observed to have high irreversible fouling (Rir), requiring chemical cleaning using ethylene diamine tetra-acetate (EDTA) to be utilized to remove foulants presence on the membranes. Noticeably, TMC modified NF (NF3–NF5) membranes showed susceptibility to irreversible fouling, as evidenced by the low FRR values. The overall irreversible fouling results indicate that fouling remains a challenge in real wastewater applications.

#### 3.6.5. SEM and EDX Spectra Analysis After Performance Studies of PES-Fe_3_O_4_/ZnO Nanofiltration Membranes

**Figure 10:**
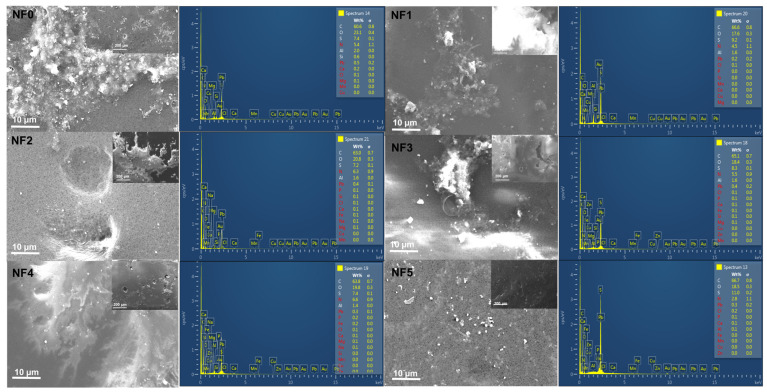
SEM images and EDX spectra of the prepared NF membranes.

Figure 10 shows SEM images of NF membranes after filtration studies using environmental wastewater samples obtained by the Spectroquant technique. The fouling degree on each membrane varied. All NF membranes had white-like crystal/cake layers on the surface of the membranes. The spots indicated that the membranes’ structural morphology had been affected by fouling, as there was no visibility of pores on the membrane surface for all membranes except for NF4 [62,63]. This further indicates that the reusability of the membranes does affect the availability of active adsorption sites; hence, fouling was observed.

**Table 3:** Comparative literature for salts and heavy metal rejections of NF membranes.

Membrane Type	Monomer Type and Concentration (*w*/*v*%)	Salt Type and Interception (%)	Heavy Metal Rejection (%)	Reference
NF3	TMC (0.10)	MgCl_2_ (12.67) Na_2_SO_4_ (50.91) NaCl (52.64)	Pb(II) (80.39, Spectroquant) Pb(II)(87.09, ICP-MS)	This study
MMT-Fe_3_O_4_/PES		NaCl (39.8) Na_2_SO_4_ (31.2) MgSO4 (39.4)	Zn^2+^ (52.5) Cu^2+^ (42.8) Ni^2+^ (35.0) Cd^2+^ (37.9)	[64]
Ag@ZnO-OAc/PA	TMC (0.15)	Na_2_SO_4_ (98.84) NaCl (59.94)		[65]
PES with citric acid as an additive		NaCl (26.83) MgSO_4_ (89.36)	**-**	[66]
Piperazine (PIP)-based polyamide (PA)	TMC (0.10) TMC (0.02)	MgCl_2_ (94.91) Na_2_SO_4_ (95.96) Na_2_SO_4_ (98.44) MgCl_2_ (92.12) NaCl (37.41)	**-**	[41]
PES/MOF	TMC (0.10)	NaCl (97.4)		[67]
Dual layer polybenzimidazole/PES			Cr^2+^ (98) Pb^2+^ (93) Cd^2+^ (70)	[68]
Polyamide (PA)		Na_2_SO_4_ (97.7)	Cu^2+^ (93.9) Mn^2+^ (97.9) Cd^2+^ (87.7)	[69]
NF90 NF270		MgSO_4_ (≥97) NaCl (85–95) MgSO_4_ (≥97) NaCl (40–60)		[70]

## 4. Adsorption Studies for the Removal of Lead (II) from Environmental Water Samples

### 4.1. Effect of pH

**Figure 11:**
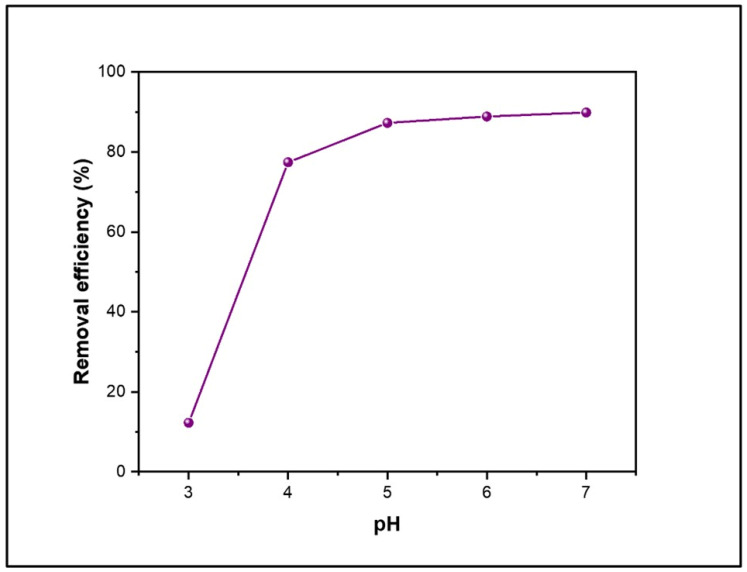
Effect of pH on Pb(II) adsorption by NF3 (PA TFC/PES-coreshell-Fe_3_O_4_/ZnO) membranes.

Generally, PES membranes are negatively charged, aiding attraction and enhancing the removal of Pb(II) ions present in environmental water samples [71], although in acidic conditions the membranes’ negative charge tends to weaken, therefore resulting in the depletion of strongly binding Pb(II) ions due to the excess hydrogen ions that compete with Pb(II) ions for binding active sites. In contrast, as the pH of the samples increases, PES membranes tend to be more negatively charged, further enhancing their adsorption of Pb(II) ions onto the surface, pores, or wall of the NF membranes [72]. Figure 11 illustrates the influence of the pH of NF3 (PA TFC/PES-coreshell-Fe_3_O_4_/ZnO) membranes on the removal of Pb(II) ions from pH 3 to 7. It was observed that the removal efficiency of Pb(II) ions increased with increasing pH, further elaborating that there was deprotonation of the excess carboxyl and hydrogen ions groups, resulting in 87.27% removal efficiency of Pb(II) at pH 5. It is noteworthy that the effect of adsorbent pH is driven by the speciation of the adsorbate. Thereafter, at pH 6 and 7, it was observed that the pH steadily increased, reaching a plateau, indicating that the availability of Pb(II) in its ionic form reduced as the speciation of Pb(II) changes with increasing pH [73]. It is well known that at pH 7 there is no speciation of lead Pb(II); instead, the presence of Pb(OH)_4_ exists at that pH [74]. Therefore, pH 5 was chosen as the optimum pH to carry out adsorption studies.

### 4.2. Effect of Concentration

**Figure 12:**
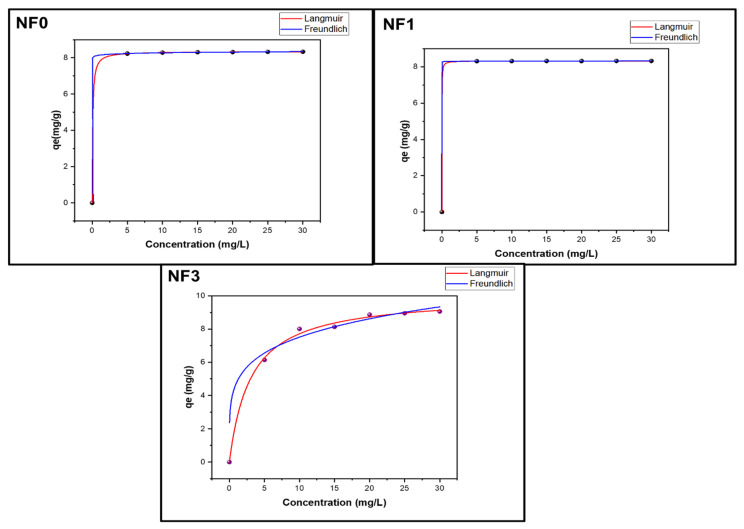
Isotherm models of lead (II) removal utilizing adsorptive NF membranes (adsorption parameters: 20 mg, pH 5, 24 h, and 25 °C).

The effect of concentration (5–30 mg/L) on Pb(II) ions removal is shown in Figure 12. The adsorption of the NF membranes was determined using both Langmuir and Freundlich isotherm models. The maximum adsorption capacity of Pb(II) ion solution for NF3 was 8.86 mg/g, indicating that the presence of modified Fe_3_O_4_/ZnO and TMC functional groups (COOH/-NH_2_) enhanced the rate of mass transfer attributed to the increased access of active sites present on the surface of the membrane. The obtained low adsorption capacity of 8.86 mg/g is adequate, considering the complex matrix environmental wastewater used, which contained high COD content, as indicated in Table 2. For pressure-driven filtration membranes with accessible Pb(II) active sites, it is demonstrated that the modified Fe_3_O_4_/ZnO nanocomposites and polyamide layer functional groups enhance the adsorption removal of Pb(II) through the availability of active sites in real complex wastewater conditions, outperforming reported adsorbents tested only using synthetic wastewater samples. This performance highlights that NF3 has practical robustness for complex real wastewater applications. All the NF membranes favored the Langmuir model. The results of the fitting isotherms are shown in Table 4. The correlation coefficient values (R^2^) indicated that the Langmuir isotherms ranged from R^2^ = 0.9905 to 0.9997 for NF0–NF3, suggesting that the Langmuir Isotherm is best suited for the adsorption of Pb(II) ions on the membranes compared to the Freundlich Isotherm model (R^2^ = 0.9076 to 0.9381). The n values for NF0, NF1, and NF3 were greater than 1, indicating favorable adsorption. NF3 was the optimal membrane that exhibited the most efficient Pb(II) adsorption capacity with monolayer adsorption dominance under the tested conditions. It is acknowledged that, in a complex wastewater matrix, adsorption involves physisorption, chemisorption, and ion-exchange. The best-fitted isotherm indicates that the Pb(II) ions adsorb on a homogenous monolayer surface with sites of similar affinity for metal ions. Conformity with the kinetic models further elaborates that the primary adsorption takes place through chemical interaction [75].

### 4.3. Effect of Time

**Figure 13:**
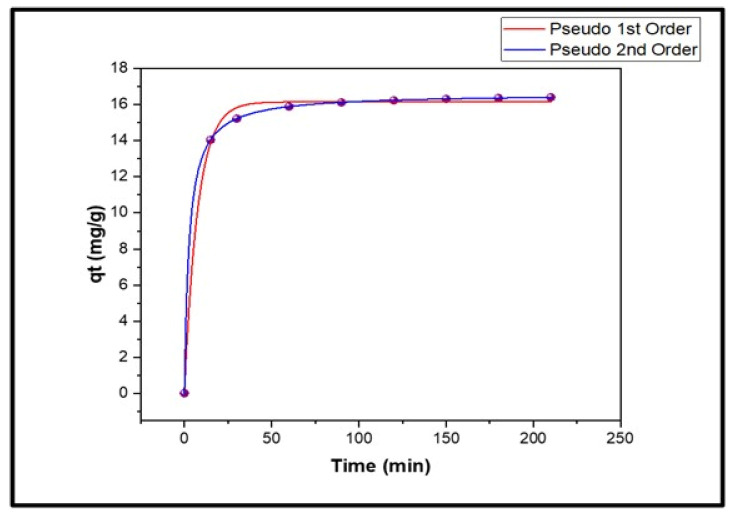
Adsorption kinetics of lead (II) removal utilizing different PA TFC/PES-coreshell-Fe_3_O_4_/ZnO nanofiltration membranes (adsorption parameters: dosage = 20 mg, pH = 5, time = 90 min and temperature = 25 °C).

The pseudo-first-order and pseudo-second-order kinetics show the insight of Pb(II) removal adsorbed by NF3 membrane [76]. The produced NF3 membrane adsorption capacity reached a removal uptake of 16.1314 mg/g at 20 mg/L and was directly proportional to contact time. As the graph illustrates (Figure 13), adsorption site saturation causes the membrane to plateau at about 90 min. First- and second-order pseudo-models were used to further analyze the kinetics isotherm data. With R^2^ values of 0.9973 and 0.9999, respectively, and qt values of 16.16 and 16.62 mg/g concurring with a similar study [77], Table 5 displays the pseudo-first- and pseudo-second-order results. The high R^2^ value indicates that the process aligns with chemisorption, entailing chemical interactions rather than physical interactions. The findings of this study concur with Maximous et al. [78], supporting chemisorption as the dominant mechanism for Pb(II) removal from natural wastewater. The Kl value of the pseudo-first-order indicates the rate of reaction (adsorption capacity). It was noticed that the K_1_ rate of reaction was below <1, indicating that there was rapid surface adsorption due to electrostatic interactions between Pb(II) ions and the active sites (amide groups, carboxyl groups, and hydroxyl groups) from the Fe_3_O_4_/ZnO nanocomposites and TMC/MPDA monomers. The K_2_ value of the pseudo-second-order indicates that the rate of reaction is close to stabilization at 90 min, as strong adsorption bonds between Pb(II) ions and active sites are formed. Additionally, NF3 best fitted the pseudo-second-order model. The increased adsorption capacity indicates that, within 90 min, the adsorption of Pb(II) removal was rapid due to available active sites; hence, a shorter time is required to adsorb Pb (Pb(II) from environmental wastewater samples to avoid the occurrence of desorption. Therefore, PES-Fe_3_O_4_/ZnO NF membranes produced using 0.1 *w*/*v*% TMC demonstrated superior adsorption performance for Pb(II) removal from environmental wastewater.

### 4.4. Potential Mechanism

**Scheme 2:**
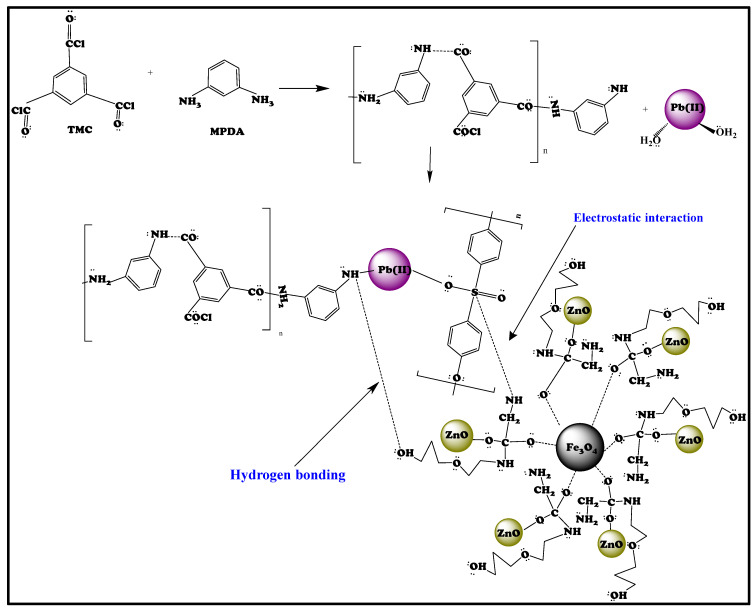
Proposed mechanism of Pb(II) adsorption from environmental water using PA TFC/PES-coreshell-Fe_3_O_4_/ZnO nanofiltration membranes.

During the formation of NF3 membranes via the IP process, TMC and MPDA reacted to form a polyamide structure through the formation of amide bonds and the release of HCl as a by-product (Scheme 2). The primary removal of Pb(II) is through the polyamide layer, which is attributed to size exclusion and Donnan effects. The produced polyamide structure has functional groups such as carbonyl and amides that coordinate with Pb(II) ions from environmental wastewater, chelating the Pb(II) ions into the polymer matrix due to the sulfonic groups of PES that enhanced the electrostatic interaction binding of Pb(II) ions. Notably, there is a presence of hydroxyl and amide-rich linkers from the embedded Fe_3_O_4_/ZnO nanocomposites of the PES NF membranes. Therefore, the novel PA TFC/PES-coreshell-Fe_3_O_4_/ZnO NF membranes (NF3) demonstrate the integrated membrane rejection and multiple active sites for the efficient binding of Pb(II) ions. This membrane is suitable for water treatment applications and can be further explored in the removal of other divalent ions.

### 4.5. Reusability and Desorption Studies

**Figure 14:**
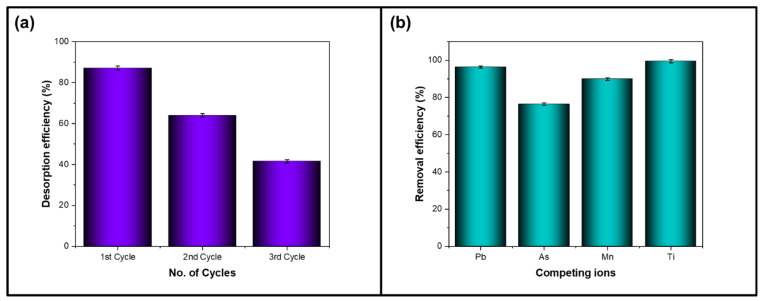
(**a**) Reusability studies of NF3 membranes for the removal of Pb(II) ions and (**b**) competing ions present in environmental wastewater.

The reusability studies in Figure 14a show exceptional removal efficiency of 89.09% after the first cycle, and there was a sufficient decrease of 63.96% and 41.49% for the second and third cycles, demonstrating that there is a decrease in the accessible active site, attributed to the decline in the potent binding affinity. The performance decline indicates the limitation in the membranes’ long-term operational durability. The observed decline can be inclusive of several factors, implying structural degradation, irreversible adsorption of captured Pb(II) ions, mechanical strength, etc. This illustrates that, while NF3 embedded active sites have strong binding capacities, the performance of the long-term durability of the NF3 membranes remains a challenge. The competing ions in Figure 14b reveal the significant differences in the selectivity of the NF3 membrane, suggesting that it has a strong selectivity of 96.31% for Pb(II), 89.87% for Mn(II), and 99.64% for Ti(IV) removal, while As(V) has the least removal efficiency of 76.36%. This suggests that NF3 membranes are governed by electrostatic interaction, speciation, and Lewis-base interactions. Therefore, the optimized parameters and added additives played a significant role in enhancing the performance of NF3, making it versatile for complex water systems. The findings of this study concur with PA NF membranes with finely tailored pores for high-performance permeance and the rejection of heavy metals and dyes [79].

## 5. Conclusions

The produced PES/coreshell-Fe_3_O_4_/ZnO nanofiltration membranes demonstrate high removal efficiencies for lead (II) ions, with rates exceeding 80% and a removal capacity of 8.86 mg/g. While this removal capacity was moderate, it demonstrates the effectiveness of NF3 membrane for complex real wastewater applications. This efficiency is maintained across a range of pH values and operational conditions, showcasing the membrane’s robustness and versatility, confirmed by SEM, AFM, FTIR, and TGA analysis. This study established that controlled TMC concentration loading is critical for engineering a selective polyamide layer TFC membrane. The combination of adsorption and filtration mechanisms allowed for the effective capture of Pb(II) ions and other competing ions, while the membrane’s structural stability due to the presence of TMC loading ensures consistent performance over extended periods of use. Overall, the integration of coreshell-Fe_3_O_4_/ZnO nanocomposite into PES membranes and its synergistic tailored TMC selective polyamide layer enhanced the multifunctional performance of TFC membranes. The fabricated TFC membranes offer a viable solution for the efficient removal of Pb(II) ions from complex water sources, favoring chemisorption. While this study focused on the effect of TMC on interfacial polymerization reaction, it did not vary MPDA, reaction time parameters, or the molecular cut-off weight of these membranes. Therefore, future work on the above-mentioned factors could be investigated to further enhance the performance of polyamide thin-film composite/PES-coreshell-Fe_3_O_4_/ZnO membranes. The successful application of the optimal NF3 membrane in real complex wastewater highlights the potential of the TMC engineering approach. Therefore, the reported findings of this study revealed the need for more investigations into TFC membranes using real environmental samples. Further research and development in this area could lead to the widespread adoption of such unique NF membranes in industrial and municipal wastewater treatment facilities, contributing to environmental protection and public health and safety.

## Figures and Tables

**Table 1:** Composition and conditions of the prepared membranes.

Membrane	PES (wt.%)	PVP (wt.%)	NMP (wt.%)	Coagulation Bath Temperature (°C)	Fe_3_O_4_/ZnO (wt.%)	Solvent Evaporation Time (EVT) (s)	MPDA (wt.%)	TMC (wt.%)
NF0	18	0	82	25	0	0	2	0.1
NF1	18	2	80	25	0	0	2	0.1
NF2	18	2	80	40	0	180	2	0.1
NF3	18	2	79.50	40	0.50	180	2	0.1
NF4	18	2	79.50	40	0.50	180	2	0.2
NF5	18	2	79.50	40	0.50	180	2	0.3

**Table 2:** Psychochemical properties of water samples collected from a wastewater treatment plant located in Gauteng.

Sampling Location	pH	Conductivity (µS/cm)	Dissolved Oxygen (%)	Chemical Dissolved Oxygen (mg/L)	TDS (mg/L)
**Biological nutrient removal**	7.09 ± 0.05	15 ± 0.09	3.26 ± 0.37	>4120 ± 8	8 ± 14

**Table 4:** Isotherm models for the adsorption of NF membranes.

	Langmuir Model				Freundlich Model		
Membranes	q_experimental_ (mg/g)	q_model_ (mg/g)	K_L_ (L/mg)	R^2^	KF (mg/g)	N	R^2^
NF0	5.48	8.32	13.3590	0.9905	0.00684	8.14551	0.9389
NF1	5.72	8.32	4.505 × 10^−7^	0.9907	0.2771	3.2577	0.9172
NF3	8.86	10.05	0.3296	0.9971	0.1982	4.7579	0.9076

**Table 5:** Kinetic model parameters of NF3 membrane.

Pseudo-First-Order				Pseudo-Second-Order		
q,_exp_(mg/g)	q,_cal_ (mg/g)	K_1_ (min^−1^)	R^2^	q,_cal_	K_2_ (g mg·min^−1^)	R^2^
16.1314	16.16	0.1305	0.9973	16.62	0.0219	0.9999

## Data Availability

Data will be made available on request and can be sourced from Nompumelelo S. M. Kubheka and Makwena J. Moloto.
